# Nondestructive analysis of urinary calculi using micro computed tomography

**DOI:** 10.1186/1471-2490-4-15

**Published:** 2004-12-13

**Authors:** Chad A Zarse, James A McAteer, Andre J Sommer, Samuel C Kim, Erin K Hatt, James E Lingeman, Andrew P Evan, James C Williams

**Affiliations:** 1 Department of Anatomy and Cell Biology, Indiana University School of Medicine, Indianapolis, Indiana (USA); 2 Molecular Microspectroscopy Laboratory, Department of Chemistry and Biochemistry, Miami University, Oxford, Ohio (USA); 3 Methodist Hospital Institute for Kidney Stone Disease, Methodist Hospital, Indianapolis, Indiana (USA)

## Abstract

**Background:**

Micro computed tomography (micro CT) has been shown to provide exceptionally high quality imaging of the fine structural detail within urinary calculi. We tested the idea that micro CT might also be used to identify the mineral composition of urinary stones non-destructively.

**Methods:**

Micro CT x-ray attenuation values were measured for mineral that was positively identified by infrared microspectroscopy (FT-IR). To do this, human urinary stones were sectioned with a diamond wire saw. The cut surface was explored by FT-IR and regions of pure mineral were evaluated by micro CT to correlate x-ray attenuation values with mineral content. Additionally, intact stones were imaged with micro CT to visualize internal morphology and map the distribution of specific mineral components in 3-D.

**Results:**

Micro CT images taken just beneath the cut surface of urinary stones showed excellent resolution of structural detail that could be correlated with structure visible in the optical image mode of FT-IR. Regions of pure mineral were not difficult to find by FT-IR for most stones and such regions could be localized on micro CT images of the cut surface. This was not true, however, for two brushite stones tested; in these, brushite was closely intermixed with calcium oxalate. Micro CT x-ray attenuation values were collected for six minerals that could be found in regions that appeared to be pure, including uric acid (3515 – 4995 micro CT attenuation units, AU), struvite (7242 – 7969 AU), cystine (8619 – 9921 AU), calcium oxalate dihydrate (13815 – 15797 AU), calcium oxalate monohydrate (16297 – 18449 AU), and hydroxyapatite (21144 – 23121 AU). These AU values did not overlap. Analysis of intact stones showed excellent resolution of structural detail and could discriminate multiple mineral types within heterogeneous stones.

**Conclusions:**

Micro CT gives excellent structural detail of urinary stones, and these results demonstrate the feasibility of identifying and localizing most of the common mineral types found in urinary calculi using laboratory CT.

## Background

Clinical laboratory assessment of urinary stones is typically conducted using destructive methods of analysis [1-5] and is usually geared to identify stones only by their primary mineral content. It is rare that stones get classified as having multiple components, and even less likely that notation is given to describe the pattern or distribution of different minerals in heterogeneous stones. Thus, stones are commonly classed as being calcium oxalate monohydrate (COM), uric acid, cystine, etc. When this information makes its way to the patient's chart, the individual may be classified simply, for example, as a COM stone former. This tends to underestimate the complexity of an individual's stone history as, indeed, it has been determined that the vast majority of stones actually contain more than one type of mineral [6].

Knowing the mineral composition of a patient's stones has obvious value in determining a treatment plan, but stone structure may be important as well. It has long been appreciated that there is variability in stone fragility to shock waves in lithotripsy, that stones of a given mineral type do not all break the same [7]. Stone fragility, and the factors that may influence variability in fragility, are not well known. There are some clues, but the story is incomplete and deserves attention. As an example, consider the case of cystine stones. Rough surface cystine stones tend to break readily, but smooth surfaced cystine can be very difficult to break [8]. Rough cystine typically contains internal radio-lucent regions (possible voids). Numerical modeling, and studies with artificial stones made to contain voids, suggests that such defects could be sites of structural weakness [9]. Thus, the internal structure of a stone may very well contribute to its fragility.

The purpose of this report is to present micro computed tomography (micro CT) as a potential method for the analysis of urinary stone composition and morphology in a nondestructive manner at very high resolution. Micro CT, which has seen considerable use as a research tool in bone biology [10], has the ability to reconstruct 2-D and 3-D images of urinary stones that allow the 3-D image of the stone to be cut and viewed in multiple planes with voxel sizes of 8–34 μm. In the present study, we observed that in addition to providing exceptional detail of the fine structure of human urinary stones, micro CT was able to differentiate six common mineral constituents using x-ray absorption (attenuation) values alone.

## Methods

Human urinary stones were obtained following percutaneous nephrolithotomy procedures or from a stone analysis laboratory (Beck Analytical Services, Indianapolis, IN). A representative collection of urinary stones of pure and heterogeneous mineral content was selected for use in this study.

### Calibrating micro CT attenuation to mineral content

Stones for this work had already been analyzed using conventional methods (microscopic, chemical and IR spectroscopic), with portion used here typically the half of the stone remaining intact after that analysis. For some stones, pre-CT analysis was completed on a cohort stone taken during the same surgical procedure. Compositions of the 11 stones used for combined micro CT and IR microspectroscopy included pure COM, pure calcium oxalate dihydrate (COD), 91% COD/9% COM, 99% COM/1% hydroxyapatite, 52% COM/48% uricite, pure uricite, pure struvite, two pure cystine stones, and two pure brushite stones.

Stones were embedded within methyl methacrylate and sliced into 1–2 mm slabs using a diamond wire saw (Well Saw, Delaware Diamond Knives, DE). A Perkin-Elmer AutoImage infrared microscope interfaced to a Perkin-Elmer Spectrum 2000 fourier transform infrared (FT-IR) spectrometer (Perkin Elmer, Shelton, CT) was used to collect infrared spectra on the flat surface of the stone slices. The spectrometer directs infrared light onto the specimen through a microscope lens, and collects reflected light onto a cooled mercury-cadmium-telluride detector. Spectra were collected in grids of 200 × 200 μm intervals, averaging 16 individual scans on each spot, yielding spectral resolution of 4 cm^-1^. Spectra for COM, COD, apatite, uric acid, struvite, cystine, and brushite were all easily identified and distinguishable from one another. Uricite and uric acid dihydrate could not be distinguished above the background noise of the spectra, and so regions are reported as uric acid.

**Figure 1:**
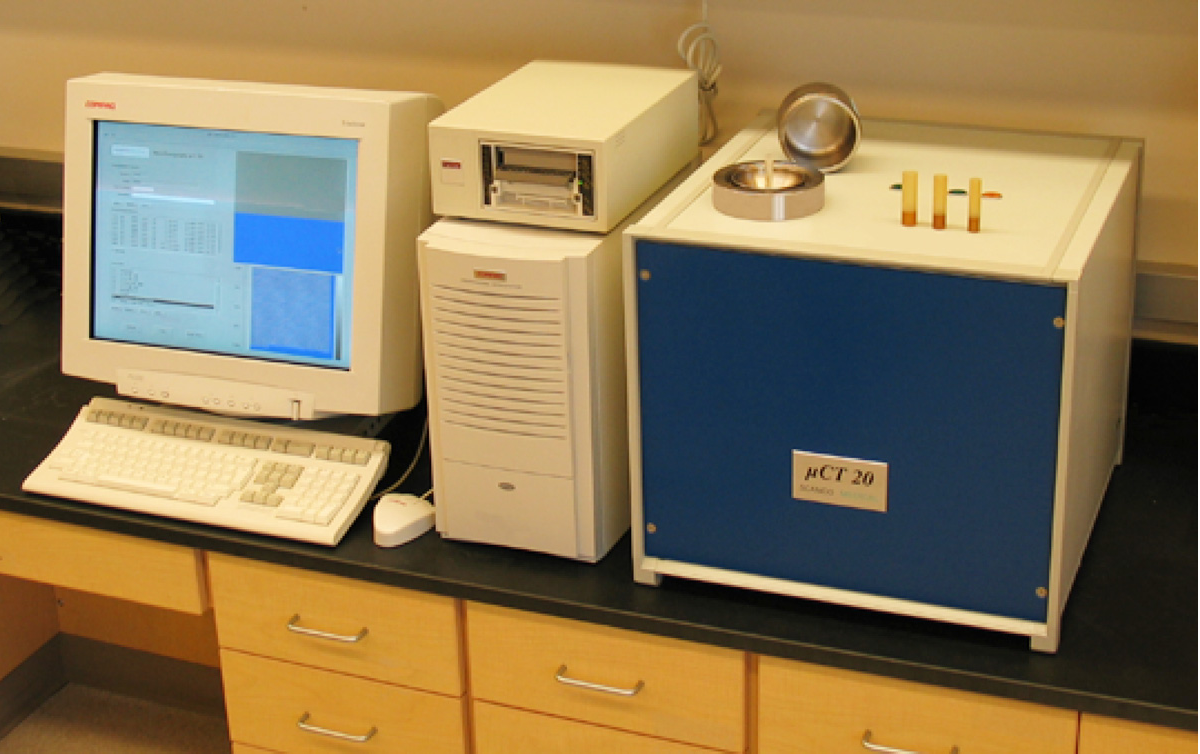
**Micro CT system (Scanco MicroCT 20)**. The micro CT unit itself is on the right, a cube approximately 0.5 m across. The computer workstation to the left of the micro CT unit controls the collection of scan data, storage and archival of data, and is used for image reconstruction.

The same stone slices analyzed by FT-IR microspectroscopy were also scanned by micro CT, using a Scanco MicroCT 20 instrument (Scanco Medical AG, Bassersdorf, Switzerland) (Figure 1). The system utilizes a 7 μm spot-size microfocus x-ray source (0.16 mA, 50 kVp) that is detected by a charge coupled device array. The scans on stone slices were completed using standard resolution (512 × 512 pixels) and a 17.4 mm specimen holder which produced image slice thicknesses and pixel widths of 34 μm. For each stone slice, a CT image slice was obtained within ~ 50 μm of the cut surface.

**Figure 2:**
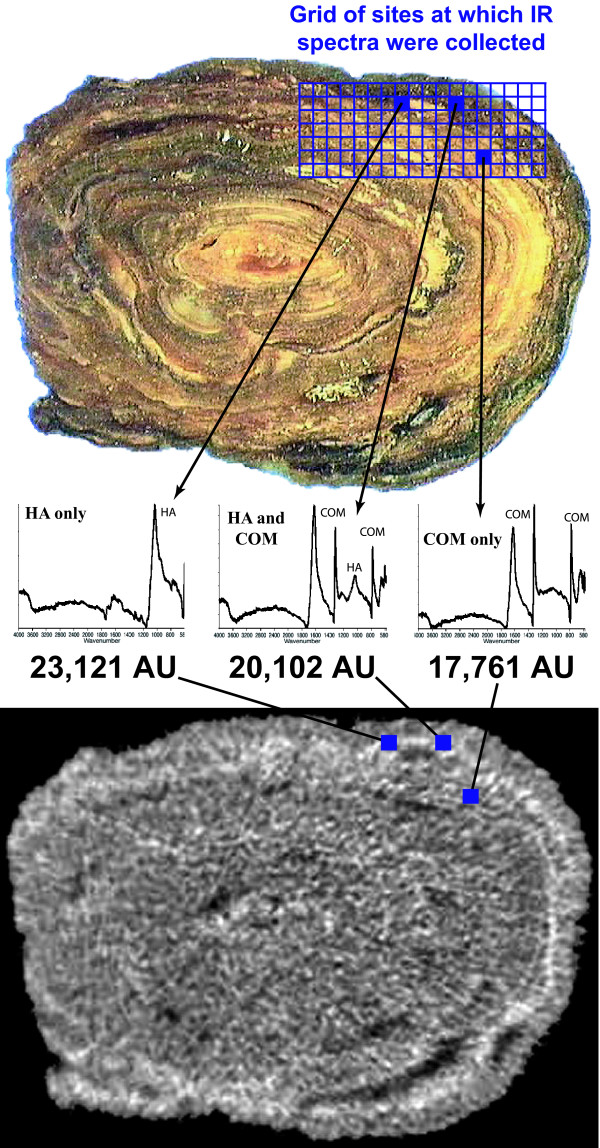
**Calibrating micro CT attenuation to pure mineral**. On stone slice shown (**top**) FT-IR spectra were collected on each of 126 regions indicated by the grid area. Examples of spectra are shown for three regions, which represent the three classes of spectra collected from cut surface of this stone. Spectra indicated that the mineral was either purely calcium oxalate monohydrate (COM), purely apatite (HA), or a mixture of these two minerals. Corresponding regions-of-interest on micro CT image slice (**bottom**) taken just beneath the cut surface are shown with blue squares, and micro CT attenuation values are given below spectra. Using this method, regions of pure mineral were identified on stone slices and corresponding regions-of-interest measured in micro CT images to determine CT attenuation of different minerals.

The FT-IR analysis yielded a 2-D map of mineral composition for a defined region of each stone slice. Regions of the 2-D map showing pure mineral were compared to the identical region on the micro CT image slice taken just below the cut surface (Figure 2). Micro CT attenuation values from these pure mineral regions were recorded.

### Imaging of intact stones: nondestructive stone analysis

In scanning intact stones with micro CT, actual slice thickness and pixel widths were dependent on the diameter of the specimen holder and resolution parameters, and ranged from 25–34 μm for images shown in this study. Examples of scans of intact stones are presented to show the potential for performing analyses of stone composition and structure using micro CT alone.

## Results

The imaging capability of micro CT is simply remarkable. Additional Data File 1 shows a stack of micro CT images of a single stone, archived as a movie. Refer to this file to view the reconstruction ability of micro CT, as well as to appreciate the power of micro CT to clearly display very high resolution images of stone structure.

**Figure 3:**
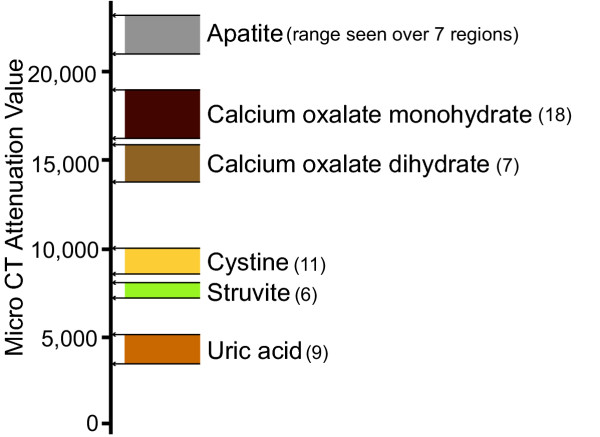
**Micro CT attenuation values taken from pure mineral regions identified by FT-IR microspectroscopy**. Regions-of-interest representing pure mineral, confirmed by FT-IR mapping, were drawn on micro CT images collected just beneath the cut surface of stone slice, and average value for CT attenuation (in machine-specific units) was recorded. Horizontal lines indicate minimum and maximum values, and number of regions-of-interest indicated in parentheses. Note that each mineral composition is associated with non-overlapping attenuation values (uric acid 3515 – 4995, struvite 7242 – 7969, cystine 8619 – 9921, calcium oxalate dihydrate 13815 – 15797, calcium oxalate monohydrate 16297 – 18449, and hydroxyapatite 21144 – 23121).

Nine sectioned stones, analyzed as shown in Figure 2, were found to contain six different mineral types by IR spectrum. Micro CT attenuation values were taken from regions of compositional homogeneity, as shown by FT-IR microspectroscopy, and Figure 3 displays the results. Values for micro CT attenuation were non-overlapping for the six minerals. Means ± standard deviations for attenuation values were 22,207 ± 709 for hydroxyapatite, 17,771 ± 837 for COM, 14,767 ± 680 for COD, 9434 ± 439 for cystine, 7633 ± 248 for struvite, and 4201 ± 564 for uric acid. Two brushite stones were also analyzed by this method, but no regions on the cut stone surface larger than about 0.5 mm across were found that were pure; spectra frequently showed intermixing of brushite with COD. Micro CT attenuation for 11 ROI's on these two stones averaged 19,145 ± 486, with a tight range of 18,540 to 19,944.

**Figure 4:**
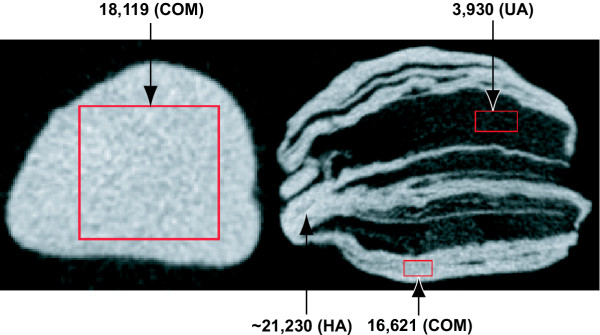
**Example of micro CT identification of mineral composition**. Visually, there appears to be only one mineral contained in the stone in the left panel and three different minerals that comprise the stone in the right panel. Micro CT attenuation identified the left stone as pure COM, and the right stone as a mixture of hydroxyapatite (bright white, highest attenuation), COM (gray) and uric acid (close to black). Speckled nature of colors – particularly apparent in image at left – is due to image noise as a result of increased magnification.

Since micro CT attenuation values for six of these minerals did not overlap, mineral composition of pure mineral regions could be inferred using micro CT alone. For example, Figure 4 displays a simple, homogeneous stone and a complex, heterogeneous stone for which micro CT attenuation values were used to identify mineral composition. The homogeneous stone (left panel) showed uniform CT attenuation values in regions-of-interest of various sizes and shapes, placed at various positions across the image, all consistent with this stone being purely COM. The image of the heterogeneous stone (right panel) displays intricate structure in which the grayscale color difference in selected regions of interest yielded different (i.e., non-overlapping) x-ray attenuation values – values that when compared to FT-IR calibration data (Figure 3) could be identified as uric acid, COM, and hydroxyapatite.

**Figure 5:**
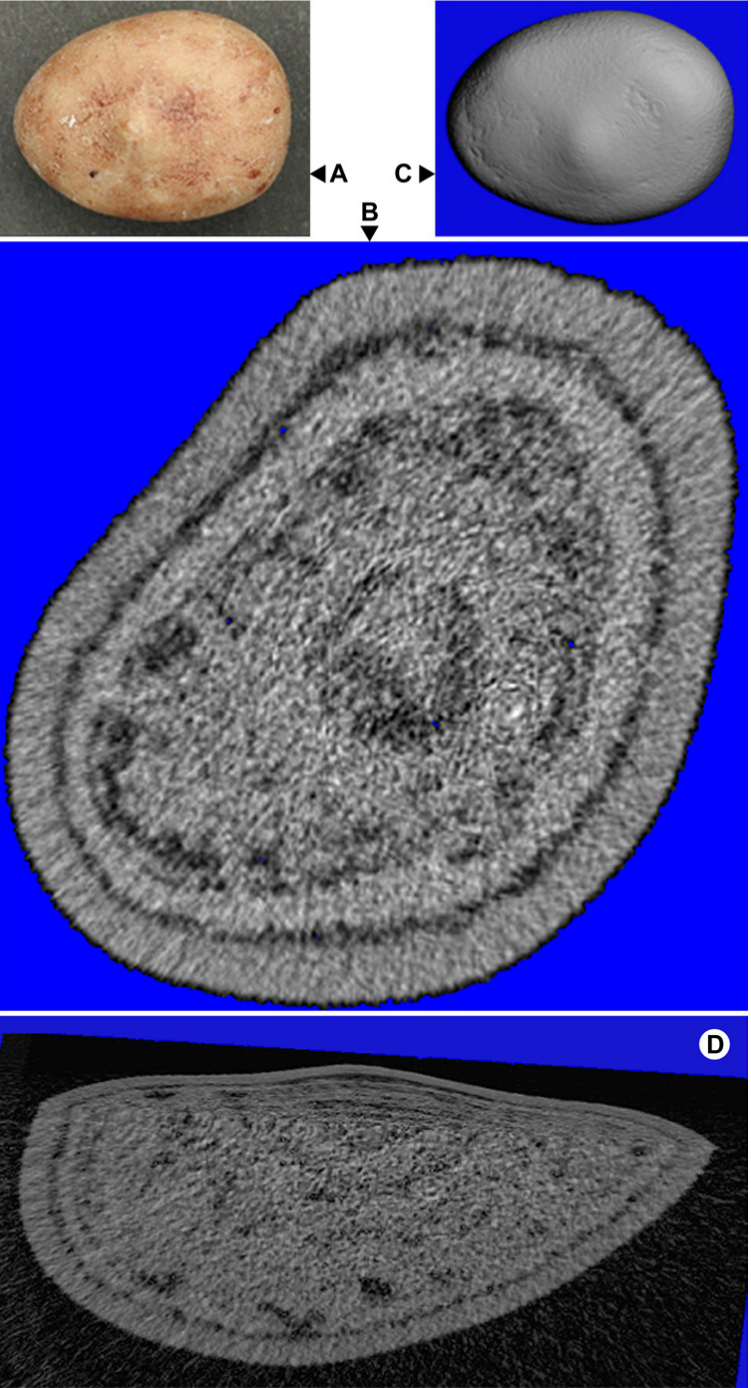
**Imaging and software power of micro CT**. Pure COM stone viewed by conventional photography (**A**) and micro CT (**B-D**). **B **shows a typical micro CT image slice, **C **a 3-D reconstruction of the stone surface, and **D **a wedge cut that displays the internal 3-D morphology of the stone.

Over the past few years, we have imaged hundreds of urinary stones using micro CT, and we have begun to learn some of the capabilities of this method that can enhance the value of the analytical information presented above. Figure 5 shows some of the imaging capabilities of micro CT software. Panel A is a photograph of a COM stone and panel B is a representative micro CT image slice in which the concentrically lamellar nature of the stone is easily seen. The dark lines separating lamellae indicate regions that absorb x-rays poorly, possibly representing regions of organic matrix. The 3-D surface rendering capability of micro CT software is shown in Figure 5C, where surface topography is shown. Further, the software permits the image to be cut and rotated in multiple planes, as shown by the wedge cut that exhibits the 3-D lamellar distribution of voids in two planes of view (Figure 5D).

**Figure 6:**
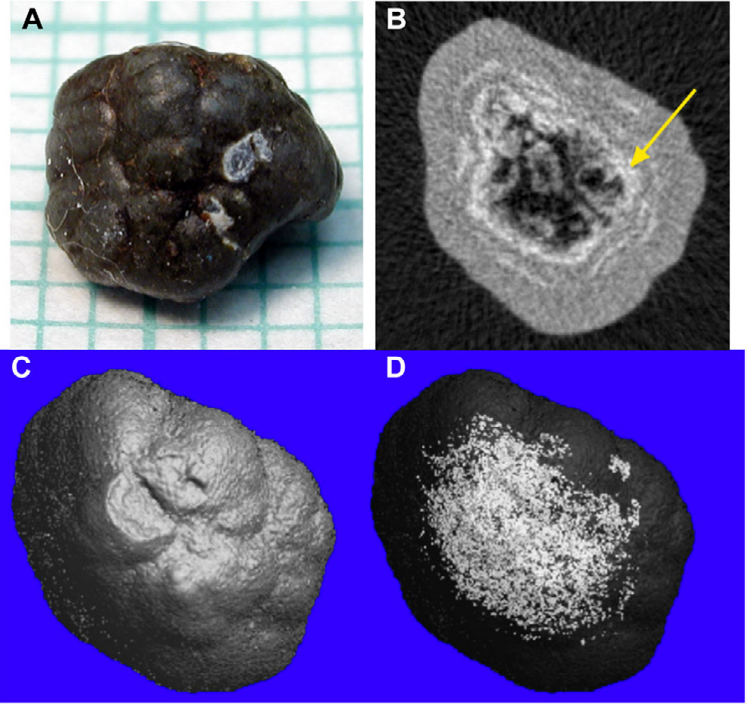
**Visualization of components of a mixed COM/HA stone**. **A. **Digital photograph of stone, on 1 mm grid. **B. **Micro CT slice through middle portion of stone; arrow indicates ring of high attenuation (hydroxyapatite). Gray material outside this ring had attenuation value in the range for COM. **C. **3-D surface rendering from micro CT displaying surface topology. **D. **3-D surface rendering with transparent shell to show 3-D localization of hydroxyapatite within stone.

Figure 6 shows features of a stone revealed using micro CT data in a different way. Panel A is a photograph of a mulberry-type COM stone and panel B is a single image slice of this stone by micro CT. The micro CT image shows a COM outer shell deep to which is a thin ring of hydroxyapatite (white) surrounding a core of low attenuation. The core consists of void spaces, which are defined to be regions of extremely low attenuation, again perhaps representing organic matter. Panel C shows a 3-D surface rendering of the stone from the micro CT image stack. Panel D displays a surface rendering of the apatite regions, superimposed within a translucent version of panel C so that the position of the apatite within the stone is easily visible. Rendering for panels C and D were obtained using MacVol, a freeware program.

**Figure 7:**
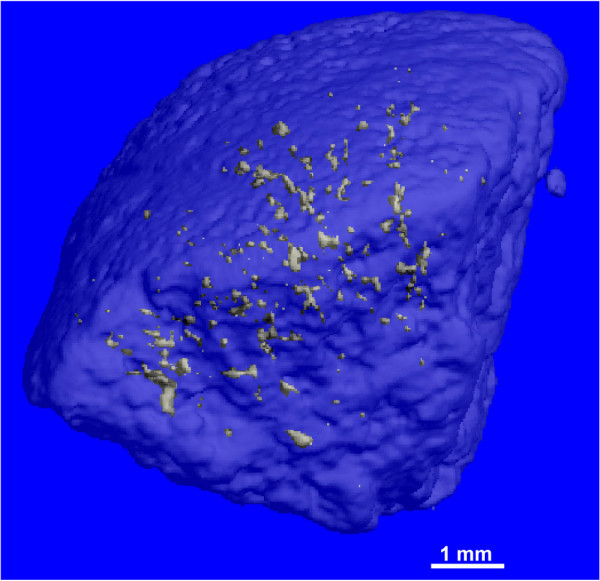
**3-D reconstruction of a cystine stone with regions of low attenuation (voids)**. Outer shell of stone was made transparent to display distribution of inner void regions depicted as small bodies. Total stone volume was 257.7 mm^3 ^and total volume of internal voids was 0.3 mm^3^.

Total stone volume and volume of internal voids can also be calculated using images obtained by micro CT. Figure 7 shows a transparent 3-D reconstruction of a cystine stone with void spaces appearing as internal granular objects. The stone volume is 257.7 mm^3 ^and the volume of internal voids is 0.3 mm^3^. This method can also be used to display and quantitate the 3-D distribution of mineral components within a complex, heterogeneous stone.

## Discussion

Micro CT yields excellent high resolution analysis of stone structure. It is a relatively fast method, taking approximately 2 hours for a complete 30 μm slice scan of a 1 cm diameter urinary stone. Micro CT allows nondestructive mapping of the internal and surface structure of urinary stones and permits identification of mineral composition based on x-ray attenuation values. Micro CT cannot differentiate mineral types when the stone is highly complex and micro-heterogeneous with significant mixing of different mineral types at a scale below the spatial resolution of the instrument. With this limitation, complete analysis for most urinary stones seems feasible using micro CT alone.

Establishing micro CT standards for complete stone analysis will require a larger data set than was obtained in the present study; for example, the measurements obtained for apatite were from only one stone that was composed mostly of COM. The x-ray attenuation of apatite was the highest of all the minerals tested, which would be expected from other CT studies [12], but the attenuation value in pure apatite stones might well be different.

It will also be important to study more brushite stones to see how this clinically important mineral can be distinguished from other minerals. Brushite stones are rare, making up only about 1% of total stones [6], but when present they are a special clinical problem, as brushite stone formers tend to form stones rapidly, and the stones are difficult to break with shock wave lithotripsy [13]. A recent study showed that less than 2% of brushite stones are pure brushite [6], but the incidence of close intermixing of mineral within brushite stones, as seen in the present study, has not been studied.

The source of variation of micro CT attenuation in stone regions that are pure by IR analysis is not apparent. Note in Figure 3 that the range of attenuation values measured for COM is greater than that seen for other minerals. This range of values could be due to varying amounts of matrix included among the COM crystals [14], or it could be due to mixing of small amounts of COD or other mineral, amounts small enough not to be detected by IR. The vast majority of stones contain more than one component [6], but many of these mixed stones show obvious spatial separation of the different materials, as observed in the stones shown in Figures 4b and 6. If a significant number of stones show close intermixing of minerals, the use of micro CT for stone analysis may be more limited than is suggested in the present study.

One application of stone analysis using micro CT will be the study of stone fragility in shock wave lithotripsy. It has been known from the earliest days of shock wave lithotripsy that some kinds of urinary stones are broken by shock waves more easily than others [11]. However, only recently has it been better appreciated that stone behavior in lithotripsy is highly variable, even among stones composed of the same major mineral type [7]. Some of this variable behavior in lithotripsy may be accounted for by the structural arrangement of stone components, which can be viewed by CT [15]. Further testing of hypotheses concerning stone structure and stone fragility can be done *in vitro*, using micro CT to analyze stones before breakage in the lithotripter.

Another logical application of micro CT is for materials testing of stones. Several studies have reported materials properties of stones [16-18], but stone heterogeneity complicates this effort. One study by Zhong et al. demonstrated that depending on the site of hardness testing within the same stone, different measurements could be obtained [19]. Thus, micro CT could help identify regions of homogeneous mineral content within a stone and also show any structural internal weakness, such as a crack or void space, prior to testing. This process would enable researchers to identify regions suitable for materials testing and, therefore, provide more reliable data.

Our findings with micro CT suggest that similar stone analysis may one day be possible as a preoperative diagnostic tool. Clinical helical CT is evolving and enhancements continue to be made to increase overall imaging functionality. For instance, newer multi-detector helical CT (MDCT) affords much greater spatial resolution than conventional single-detector helical CT. Williams et al. used four-row MDCT to show that some degree of internal structure can already be seen in urinary stones [15]. Zarse and colleagues also used four-row MDCT and demonstrated that CT can identify mineral composition *in vitro *when suggested scanning parameters were used [20]. Eight-row MDCT has been found to improve z-axis resolution and scan time, while reducing artifact streaking for an overall improvement in diagnostic imaging [21]. Sixteen-row MDCT instruments are now available that use isotropic imaging, the same technology used in micro CT. This essentially translates to equal resolution and voxel size in any plane (sagittal, coronal, and axial) [22]. Moreover, like micro CT, MDCT has the option to reconstruct 3-D images of the entire viewing area. This is advantageous since mapping the 3-D spatial distribution of mineral content in stones could yield important information useful in determining proper treatment for the patient. Overall, the continued development of helical CT technology points to imaging capability in the future that could provide stone analysis equivalent to what we describe for micro CT.

## Conclusions

Micro CT provides high resolution *in vitro *imaging of urinary calculi for nondestructive stone analysis. Fine resolution coupled with the 2-D and 3-D reconstruction capabilities of micro CT yields an imaging diagnostic that offers excellent images of surface and internal stone structure. Mineral deposition pattern and regions of potential structural weakness, such as voids, were easily visible. Six common stone minerals were found to occupy non-overlapping ranges of attenuation value, which allows the identification of mineral types using micro CT alone. This technology carries the potential for immediate application to non-destructive analysis of stone structure and composition in clinical stone analysis laboratories. The demonstration that stone composition can be determined by micro CT is proof of concept and an important step toward the use of helical CT to provide similar analysis in patients.

## Competing interests

The author(s) declare that they have no competing interests.

## Authors' contributions

CZ did the experimental work for calibrating the attenuation values, put together several of the figures, including the image stack movie, and drafted the manuscript. AJ performed the FT-IR analyses. EH did many of the micro CT scans and managed the library from which stones were chosen. SK and JL provided some of the stones from patients, and SK performed the 3-D imaging of cystine stone. JM, JL, and AE initiated much of the intellectual direction for the study. JM and AE also played important roles in the FT-IR analysis part of the study. JW performed the 3-D image construction in MacVol, drafted several of the figures, and performed the primary editing of the manuscript. All authors read and approved the final manuscript.

## Pre-publication history

The pre-publication history for this paper can be accessed here:



## Supplementary Material

Additional File 1 Micro CT image stack through a urinary stone.MOV 6.61 MB Click here for file
